# Baseline growth kinetics of pulmonary metastases in head and neck adenoid cystic carcinoma: analysis of correlated clinical and molecular factors

**DOI:** 10.3389/fonc.2026.1811232

**Published:** 2026-10-12

**Authors:** Hong-fei Liu, Ling-yan Jiang, Ting-yao Ma, Yue Zhao, Tian-yi Li, Xue-lian Wang, Shu-jing Zhang, Guo-liang Yang, Jing Zhou, Zhong-yu Yan, Xiao-hong Chen

**Affiliations:** 1 Department of Otolaryngology-Head and Neck Surgery, Beijing Tongren Hospital, Capital Medical University, Beijing, China; 2 Department of Otorhinolaryngology, Beijing University Of Chinese Medicine Third Affiliated Hospital, Beijing, China; 3 Department of Radiology, Beijing Tongren Hospital, Capital Medical University, Beijing, China

**Keywords:** adenoid cystic carcinoma, distant metastasis, growth rate, lung metastasis, single-cell sequencing

## Abstract

**Objective:**

To establish baseline growth rates of lung nodules in untreated head and neck adenoid cystic carcinoma(ACC) patients and analyze associated clinical and molecular factors influencing these rates.

**Methods:**

CT was used to calculate the diameter of 74 pulmonary metastatic nodules in 37 patients under natural state. The diameter growth rate of lung metastatic nodules was fitted with a linear regression equation, and the differences in the progression rate of lung metastasis among patients were compared. Additionally, clinical features such as location, pathological subtypes, and staging were also collected to compare their correlation with the growth rate of lung metastatic nodules. Subsequently, single-cell sequencing data from three lung metastatic nodules of differing diameters within the same case were analyzed.

**Results:**

We defined three different states based on the progression of lung metastases: rapid progression was defined as a monthly diameter increase of more than 1 mm in 16.2% (6/37) of the patients; slow progression was defined as a monthly diameter increase between 0.3-1 mm in 56.8% (21/37) of the patients; and stable state was defined as a monthly diameter increase of less than 0.3 mm in 27.0% (10/37) of the patients. In addition, factors such as low expression of TP63, a higher pathological grade (Grade III), and a nodule diameter greater than 10mm at initial diagnosis of lung metastasis were identified as significant predictors for a faster diameter growth rate (P<0.05). Subsequently, through single-cell sequencing analysis, it was found that rapidly progressing nodules exhibited upregulation of signals related to hypoxia, vascular genesis, and a weakening of immune response-related signals.

**Conclusion:**

The findings of this study suggest that baseline characteristics based on the growth rate of pulmonary metastatic nodules may be used to help stratify the progression rate of lung metastasis. In particular, based on our observations, for patients with pulmonary metastatic lesions presenting with a nodule diameter > 10 mm, pathological grade III, and low TP63 expression at initial diagnosis, clinicians might consider adopting closer follow-up monitoring or potentially initiating intervention measures earlier. Additionally, for rapidly growing pulmonary metastatic nodules, it may be worth considering targeted therapy against hypoxic/anti-angiogenic pathways or combination with immunotherapy aimed at restoring or enhancing local immune responses.

## 1 Introduction

Adenoid cystic carcinoma (ACC) of the head and neck is a notorious rare malignancy, accounting for approximately 1% of all head and neck malignant tumors (1). It is characterized by prominent biological features including high invasiveness, a propensity for perineural spread, and distant metastasis at advanced stages (2),with the lung being the most common site of metastasis (approximately 70% of patients with metastasis have lung involvement) (3). Although ACC grows relatively slowly, the development of lung metastasis often portends a poor prognosis. Studies have reported that the 5-year, 10-year, and 20-year overall survival rates for patients without distant metastasis are 85.6%, 67.4%, and 50.4%, respectively, whereas those for patients with distant metastasis drop to 69.1%, 45.7%, and 14.3% respectively (4).

Currently, there are several controversies regarding the treatment of ACC with lung metastasis as follows:(1)Absence of a stratification framework and aligned therapeutic approaches for individuals post-diagnosis of lung metastasis: Currently, effective comprehensive treatment options for ACC with lung metastasis are scarce. In several clinical trials of ACC summarized by Larissa Di Villeneuve et al., the maximum objective response rate was only 0.7% (5). A study by Zhu et al. using Apatinib reported a better objective response rate (ORR 46.2%), but compared to the average survival of 32.3-33 months post ACC distant metastasis, no significant long-term survival benefits were observed (overall survival rate at a mere 24 months stands at 82.3%) (6–8). Hence, the primary focus for therapeutic intervention ought to be high-risk lung metastasis patients showing rapid progression and limited survival. Yet, at present, there’s an absence of risk stratification approaches grounded on the speed of lung metastasis progression. (2) Ambiguous treatment intervention points: Unlike distant metastases in other organs, ACC lung metastases generally manifest a prolonged and slow progression without evident symptoms (7, 8). Consequently, there’s an ongoing debate about the appropriateness of adopting an active treatment protocol for early stable nodules that show no marked progression. (3) Deficiency in suitable evaluation indicators for lung metastasis inhibitory effect: Distant metastases in patients with ACC predominantly occurs in the lungs, and approximately 10% of these patients endure a survival period of exceeding 10 years (8). Therefore, elucidating the growth kinetic characteristics of lung metastases, namely their growth rate and pattern at baseline, is of critical significance for formulating individualized treatment strategies (such as determining the optimal timing for surgery and selecting targeted interventions) and evaluating prognosis. Meanwhile, owing to pulmonary metastatic lesions in ACC patients generally progress steadily, and most metastatic nodules take several years to develop obvious volumetric enlargement. Therefore, we adopted linear regression to simulate the growth kinetics of metastatic lesions instead of traditional evaluation indicators such as tumor volume doubling time, which enables clinicians to rapidly assess the progression status of lesions during routine clinical follow-up.

Existing studies have shown that the clinical course of lung metastasis in head and neck ACC is characterized by significant heterogeneity: some patients exhibit indolent growth of metastases and can survive with tumors for a long time; others show rapid progression, with multiple metastases or organ failure occurring in a short period. Reported potential clinically relevant factors include: primary tumor site, TNM stage, and pathological grade (9, 10). However, most current studies are limited to single-center, small-sample analyses and lack systematic quantitative analysis of baseline growth kinetics (such as quantification of growth rates).Molecular biology studies have shown that the malignant progression of ACC is closely associated with specific genetic alterations, such as MYB-NFIB gene rearrangement (11), activation of the PI3K/AKT pathway, TP53 mutation, etc. (12). These molecular events may affect the growth behavior of metastases by regulating cell proliferation, invasion, and angiogenesis. However, existing studies rarely directly link molecular markers to the growth kinetics of metastases, and there is a particular lack of analyses on the molecule-phenotype correlations specific to lung metastases of head and neck ACC.

In recent years, among molecular targeted agents, multi-targeted kinase inhibitors against vascular endothelial growth factor receptors (VEGFRs) have emerged as the most widely studied systemic therapeutic strategy for recurrent and metastatic ACC (13).Among these agents, VEGFR-TKIs constitute the core research focus. They exert anti-tumor effects by suppressing the VEGFR signaling pathway, thereby inhibiting tumor angiogenesis and modulating hypoxia-associated signaling cascades (14). VEGFR inhibitors have demonstrated definite antitumor activity in ACC, with a tumor response rate (TRR) of 67% and a disease stabilization rate of 82%. Such favorable disease control is durable, and the 6-month disease control rate (DCR) reaches 54% (15). However, most existing studies mainly focus on overall therapeutic efficacy, without precisely screening potential beneficiaries based on baseline characteristics such as the growth rate of pulmonary metastatic nodules and TP63 expression levels.

To demonstrate deeper into this matter, we undertook a retrospective analysis of 37 patients to study the progression rate of 74 nodules in their natural state for patient stratification. Additionally, we analyzed single-cell sequencing data from three lung metastatic lesions with varying progression rates within the same case to compare molecular expression differences and features of the progression patterns. The findings of this study are expected to provide new theoretical basis for prognostic stratification and precision treatment of lung metastases in head and neck ACC.

## 2 Method

### 2.1 Clinical information collection

This study conducted a retrospective analysis of clinical data from patients with adenoid cystic carcinoma who sought medical attention at Beijing Tongren Hospital from September 2000 to January 2021. The clinical information included gender, age, primary site, T stage, N stage, M stage, clinical stage, pathological grade, TP63, Ki67, diameter of pulmonary nodules, and time of lung metastasis. The pathological diagnosis of adenoid cystic carcinoma in all primary sites was confirmed by two or more pathologists. Pathological grading followed Szanto’s scheme (16). Tumor staging of primary sites adhered to the eighth edition of the American Joint Committee on Cancer (AJCC) guidelines. All non-contrast chest CT scans in this study were performed with a unified standardized protocol: tube voltage of 120 kVp, tube current ranging from 150 to 240 mA, reconstruction slice thickness of 1.0 mm and reconstruction interval of 0.5 mm. All images were reconstructed using standard lung window algorithms. The radiological diagnostic criteria for lung metastatic nodules involve an increase in their diameter during the follow-up period (with at least two or more chest CT scans, with an interval of more than three months between scans), and confirmation by two or more experienced radiologists specialized in imaging interpretation. This study was approved by the Ethics Committee of Beijing Tongren Hospital(ethics number: TREC2022-KY023).

Detailed inclusion and exclusion criteria

Inclusion criteria:

1. Pathologically confirmed head and neck adenoid cystic carcinoma;
2. Confirmed pulmonary metastasis via chest contrast-enhanced CT;
3. Complete serial chest CT imaging data with clear measurable pulmonary metastatic nodules;
4. Sufficient follow-up data to calculate natural growth rate of lesions.

Exclusion criteria:

1. Received systemic chemotherapy, targeted therapy, immunotherapy or other systematic anti-tumor treatments before the last untreated imaging time point;
2. Received pulmonary lesion resection, radiotherapy, ablation and other local treatments for lung metastases;
3. Combined with other malignant tumors;
4. Incomplete imaging data or unmeasurable metastatic nodules.

### 2.2 Continuous monitoring and analysis of pulmonary metastatic nodules

Under natural conditions, the follow-up time for pulmonary metastatic nodules is defined as the interval from the initial discovery of lung metastasis to the last chest CT scan, without receiving systemic treatment interventions (chemotherapy, targeted therapy, surgery, and other treatments). We collected the results of successive chest CT scans, measured the diameter of pulmonary nodules, and calculated the diameter growth rate. The calculation method for diameter growth rate involves recording the diameters of pulmonary metastatic nodules on two or more occasions, along with the corresponding time points. The linear regression equation, y = kx + b, is used to fit the growth pattern, where the slope (k) represents the rate of diameter growth.

Here are two measurement schemes (**Figure 1**): (1) Maximum Diameter Single Nodule Measurement: select the nodule with the maximum diameter for each patient at the initial diagnosis(defined as major nodule), locate according to the specific location of the lesion, record and analyze the growth change of the major nodule in subsequent monitoring, and analyze the growth rate of diameter; (2) Comprehensive Analysis of Multiple Nodules: Original images from patients presenting with multiple lesions were scanned, and the CT images were integrated into an AI-driven diagnostic platform (InferRead CT Lung, Infervision Medical Technology) for automated nodule detection. Nodules flagged by the InferRead system underwent independent verification by two radiologists with expertise in thoracic imaging.

**Figure 1:**
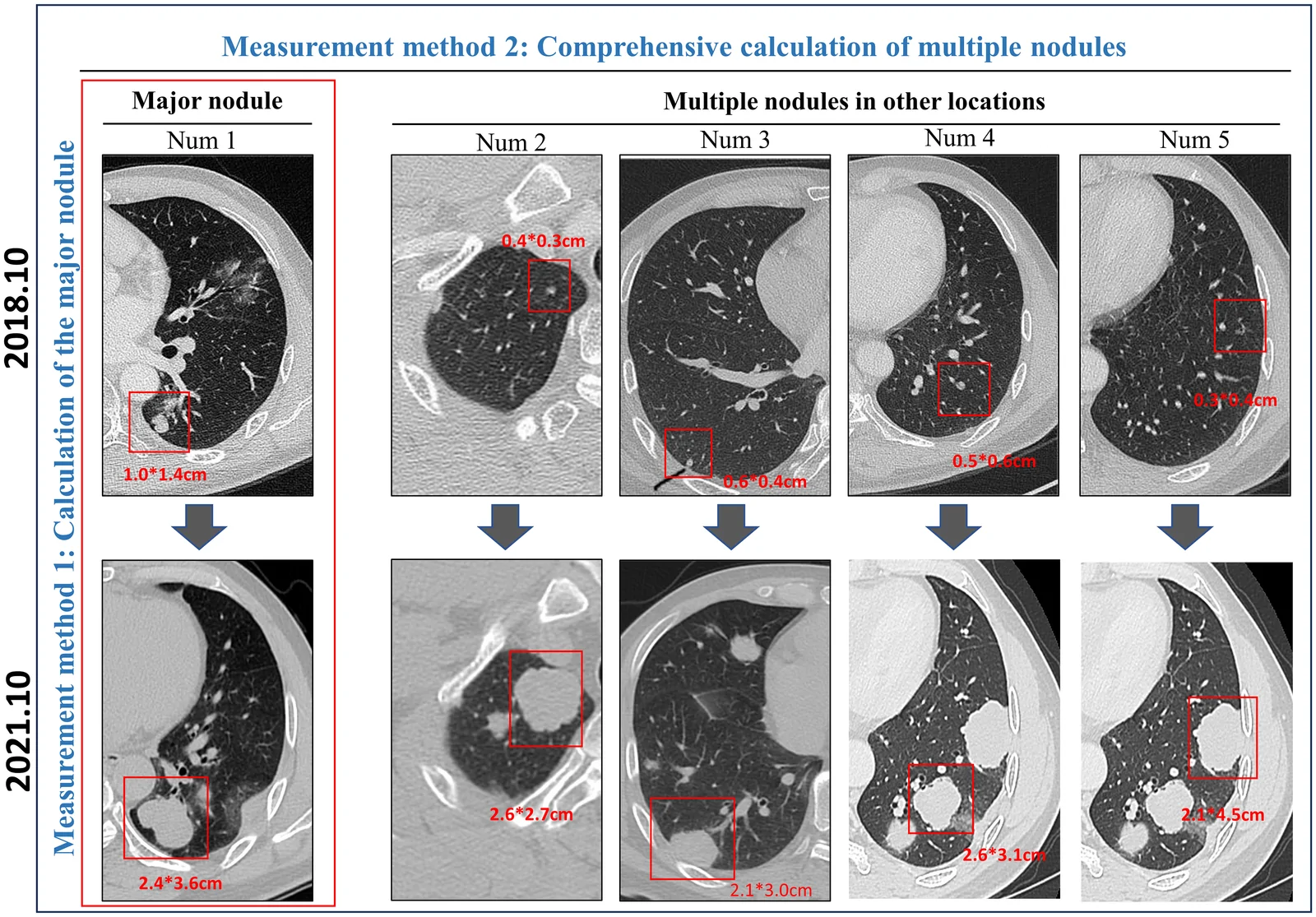
Two methods for measuring the rate of progression of lung metastasis (1) Primary nodule measurement method: This method involves measuring the largest diameter of the lung metastatic lesion (dominant nodule) at the time of initial diagnosis of lung metastasis. Continuous monitoring of the primary nodule is conducted. (2) Comprehensive measurement method for multiple nodules: This method involves measuring multiple lung metastatic lesions within the same case. Continuous monitoring is performed, and the average and median values of the measurement parameters are compared.

### 2.3 Single-cell sequencing data analysis

The single-cell sequencing data was extracted from our team’s previously published research (GEO number: GSE216852). The specific methods are as follows: Total RNA extracted from 1 × 10^7^ cells in 1 mL of TRIZOL reagent was sent to BGI for RNA-seq analysis. Total cellular RNA was extracted using an RNA extraction kit and reverse transcribed into cDNA using a reverse transcription kit. Total RNA concentration, RNA integrity number (RIN), 28S/18S ratio, and fragment size were assessed using an Agilent 2100 Bioanalyzer. All samples meeting the quality criteria were used for library construction and sequencing. BGI performed sequencing using Illumina technology and developed the Dr. Tom system for subsequent sequencing analysis.

We collected single-cell sequencing data from three lung metastatic nodules and their corresponding normal lung tissues from the same patient. The samples were sorted based on their diameter as follows: (1) Right middle lobe nodule B1 (0.4 cm, 13,257 cells); (2) Right lower lobe nodule A1 (1.3 × 1 × 0.5 cm, 7,950 cells); (3) Right lower lobe nodule C1 (1.5 × 1 × 0.7 cm, 7,729 cells). Additionally, the normal lung tissue(F) consisted of 8,014 cells. The analysis parameters were the same as in the previous study (17). The single-cell sequencing data were reanalyzed using R software version 4.2.3, and pseudotime analysis was performed using the monocle2 package.

## 3 Results

### 3.1 The overall clinical characteristics of the population and the features of pulmonary metastasis

This study encompassed a total of 37 patients with pulmonary metastasis of ACC. The average follow-up time was 55.97 months (with a median follow-up time of 37.00 months). The average duration of monitoring pulmonary metastatic nodules was 15.24 months (with a median of 12.02 months). Among them, males accounted for 27.03% (10/37), while females accounted for 72.97% (27/37). The mean age was 42.84 years (with a median age of 42 years)., there were 23 patients aged < 50 years (62.16%) and 14 patients aged ≥ 50 years (37.84%).The major primary site was the major salivary glands, accounting for 35.14% (13/37). The secondary sites were as follows: nasal cavity and sinuses accounted for 27.03% (10/37), larynx and cervical trachea accounted for 16.22% (6/37), oral cavity and oropharynx accounted for 13.51% (5/37), and other sites accounted for 8.11% (3/37). The distribution of tumor staging was as follows: T1-T2 accounted for 29.73% (11/37), while T3-T4 accounted for 70.27% (26/37); N0 staging accounted for 86.49% (32/37), while N+ accounted for 13.51% (5/37); M0 was observed in 78.38% (29/37) of patients, while M1 was observed in 21.62% (8/37). Clinical staging was categorized as follows: stages I-II accounted for 24.32% (9/37), and stages III-IV accounted for 75.68% (28/37). The main treatment approach for the primary site was surgery followed by postoperative radiation therapy, which accounted for 64.86% (24/37) of cases. Some cases received surgery alone, accounting for 13.51% (5/37). Pathological grading was predominantly Grade II, accounting for 54.05% (20/37), followed by Grade I at 13.51% (5/37), and Grade III at 10.81% (4/37). Pathology information was not available for 21.62% (8/37) of the patients. Regarding Ki-67 proliferation index, 19 (51.35%) patients had Ki-67 <20%, 9 (24.32%) had Ki-67 of 20%-30%, and 3 (8.11%) had Ki-67 ≥30%, with data unavailable in 6 (16.22%) cases. For TP63 expression, 25 (67.57%) patients showed TP63 (+), 10 (27.03%) showed TP63 (++), and 2 (5.41%) had missing TP63 data (**Table 1**).

**Table 1:** Gross clinical features of the patient.

Clinical characteristic		Num(%)
**Sex**	Male	10(27.03)
	Female	27(72.97)
**Age**	<50	23(62.16)
	≥50	14(37.84)
**Primary Site**	Nasal cavity and sinuses	10(27.03)
	Major salivary glands	13(35.14)
	Oral cavity and oropharynx	5(13.51)
	Larynx and cervical trachea	6(16.22)
	Other	3(8.11)
**T stage**	T1-T2	11(29.73)
	T3-T4	26(70.27)
**N stage**	N0	32(86.49)
	N1	3(8.11)
	N2	2(5.41)
**M stage**	M0	29(78.38)
	M1	8(21.62)
**Clinical stage**	I	4(10.81)
	II	5(13.51)
	III	10(27.03)
	IV	18(48.65)
**Treatment of primary site**	Surgery	5(13.51)
	Surgery + radiotherapy	24(64.86)
	Other	8(21.62)
**Pathology classification**	Grade I	5(13.51)
	Grade II	20(54.05)
	Grade III	4(10.81)
	unknow	8(21.62)
**Ki67**	<20%	19(51.35)
	20%-30%	9(24.32)
	≥30%	3(8.11)
	NA	6(16.22)
**TP63**	+	25(67.57)
	++	10(27.03)
	NA	2(5.41)

The average time of occurrence of pulmonary metastasis in the enrolled patients was 40.73 months (with a median time of 26.00 months). The major nodule alone was monitored in 24 patients. Multiple nodules were monitored in 13 patients. Within the patient cohort, 40.5% (15/37) had pulmonary metastasis detected within 1 year after the primary diagnosis, while 21.6% (8/37) were diagnosed with pulmonary metastasis between 12-36 months. For 37.8% (14/37) of patients, the diagnosis of pulmonary metastasis occurred more than 36 months after the primary diagnosis. In 21.62% (8/37) of patients, pulmonary metastasis was detected at the time of the primary diagnosis. Among 18.9% (7/37) of patients, the maximum diameter of pulmonary nodules at the time of metastasis diagnosis was less than or equal to 5mm, while 54.1% (20/37) had a maximum diameter of 5mm-15mm, and 27.0% (10/37) had a maximum diameter greater than 15mm (**Table 2**).

**Table 2:** Overall pulmonary metastasis characteristics.

Characteristics		Num(%)
Lung metastatic free survival	<12 month	15(40.5%)
	12-36 month	8(21.6%)
	>36 month	14(37.8%)
Lung metastatic status at diagnosis of primary site	Yes	8(21.6%)
	No	29(78.4%)
Maximum diameter nodules at first diagnosis of pulmonary metastasis	≤5 mm	7(18.9%)
	5 mm-15mm	20(54.1%)
	>15mm	10(27.0%)

### 3.2 Calculation and analysis of the rate of progression of pulmonary metastasis under natural conditions

Lung metastasis nodules were identified in 37 patients, totaling 74 nodules. We analyzed the pulmonary metastases nodules in the patients in two ways. 1) Comparison of the major nodule at the time of diagnosis of lung metastases. 2) Comprehensive comparison of multiple pulmonary metastases nodules. As shown in **Figure 1**.

The growth rate of the major nodule is calculated based on our computations (**Figure 2A**). The average monthly growth rate of pulmonary nodules was determined to be 0.65mm/month (median of 0.48mm/month). The observed rate of lung metastasis progression varied among patients. Specifically, 16.2% (6/37) of patients exhibited a diameter growth rate exceeding 1mm/month, suggesting a rapid progression phase. Meanwhile,56.8% (21/37) had a growth rate between 0.3-1mm/month, indicating a slow progression phase and 27.0% (10/37) have a growth rate less than 0.3mm/month, indicating a stable phase (**Figure 2B**).

**Figure 2:**
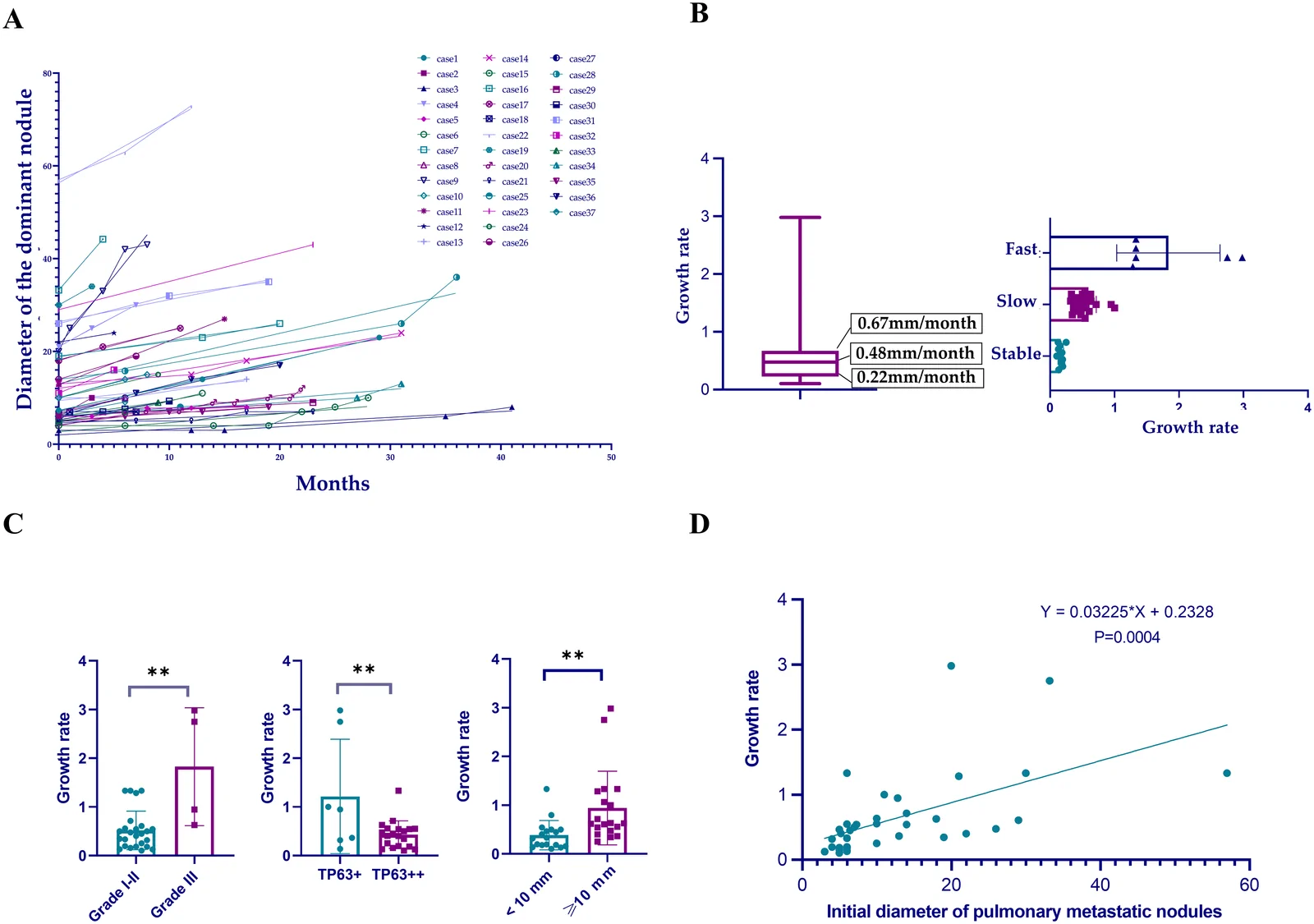
The stratification of population and risk factors for the growth rate of the primary nodule diameter in lung metastasis. **(A)** shows the line graph of the main nodule diameter growth in 37 patients. **(B)** displays the population distribution based on the growth rate of the major nodule diameter. **(C)** presents the risk factors for diameter growth rate. **(D)** illustrates the correlation analysis between the initial diameter of lung metastatic nodules and their diameter growth rate.

Performing a correlation analysis between clinical pathological features (including gender, age, primary site, T stage, N stage, M stage, histological grade, TP63, the maximum diameter of lung metastatic nodules at initial diagnosis, and time of lung metastasis occurrence) and the growth rate of lung metastatic nodule diameter was conducted (**Supplementary Table 1**). Notably, factors such as low expression of TP63, a higher pathological grade (Grade III), and a nodule diameter greater than 10mm at initial diagnosis of lung metastasis were identified as significant predictors for a faster diameter growth rate (P<0.05, **Figure 2C**). Additionally, a positive correlation was found between the initial diameter of lung metastatic nodules and their diameter growth rate (P<0.05, **Figure 2D**).

The progression of multiple pulmonary nodules in 13 patients was continuously monitored and recorded (**Table 3**). Data from various lung CT scans of individual cases with multiple nodules was analyzed, such as case 10 in **Figure 1** and **Figure 3A**. Among them, 15.3% (2/13) of cases were initially diagnosed with a single nodule, while 84.6% (11/13) had multiple nodules. On average, each patient was monitored for 4 nodules (range: 2-7), with an average of 3 measurements taken (range: 2-7) at an average interval of 8.80 months. The mean diameter of the major nodule was 15.9 mm (median: 13.0 months), with a mean diameter growth rate of 0.59 mm/month (median: 0.54 mm/month). We conducted a continuous analysis of multiple lung CT scan data from the same case with multiple nodules, as presented in Case10 shown in **Figure 1** and **Figure 3A**.

**Table 3:** Progression of multiple pulmonary nodules in 13 patients.

Case	Initial pulmonary nodules status(Num)	Initial lung nodule diameter (mm)	Total number of pulmonary nodules	The times of the measurement	Mean interval time(Mon)	Average diameter growth rate(mm/Mon)
1	Single (1)	3	7	5	10.25	0.5
2	Multiple(5)	7.6-12.8	5	2	15.00	0.78
3	Multiple(2)	21.0-22.0	2	2	5.00	0.40
4	Single (1)	13.0	2	4	10.33	0.25
5	Multiple(2)	2.0-4.0	4	7	4.67	0.32
6	Multiple(4)	12.0-18.0	4	3	3.67	0.81
7	Multiple(3)	5.8-30.0	3	2	3.00	1.38
8	Multiple(2)	46.0-57.0	2	3	6.00	1.08
9	Multiple(5)	3.0-14.0	5	2	7.00	0.57
10	Multiple(5)	4.0-14.0	5	4	12.00	0.68
11	Multiple(2)	3.0-6.0	2	2	23.00	0.45
12	Multiple(5)	3.1-6.0	5	4	6.67	0.52
13	Multiple(4)	5.1-6.4	4	2	6.00	0.57

**Figure 3:**
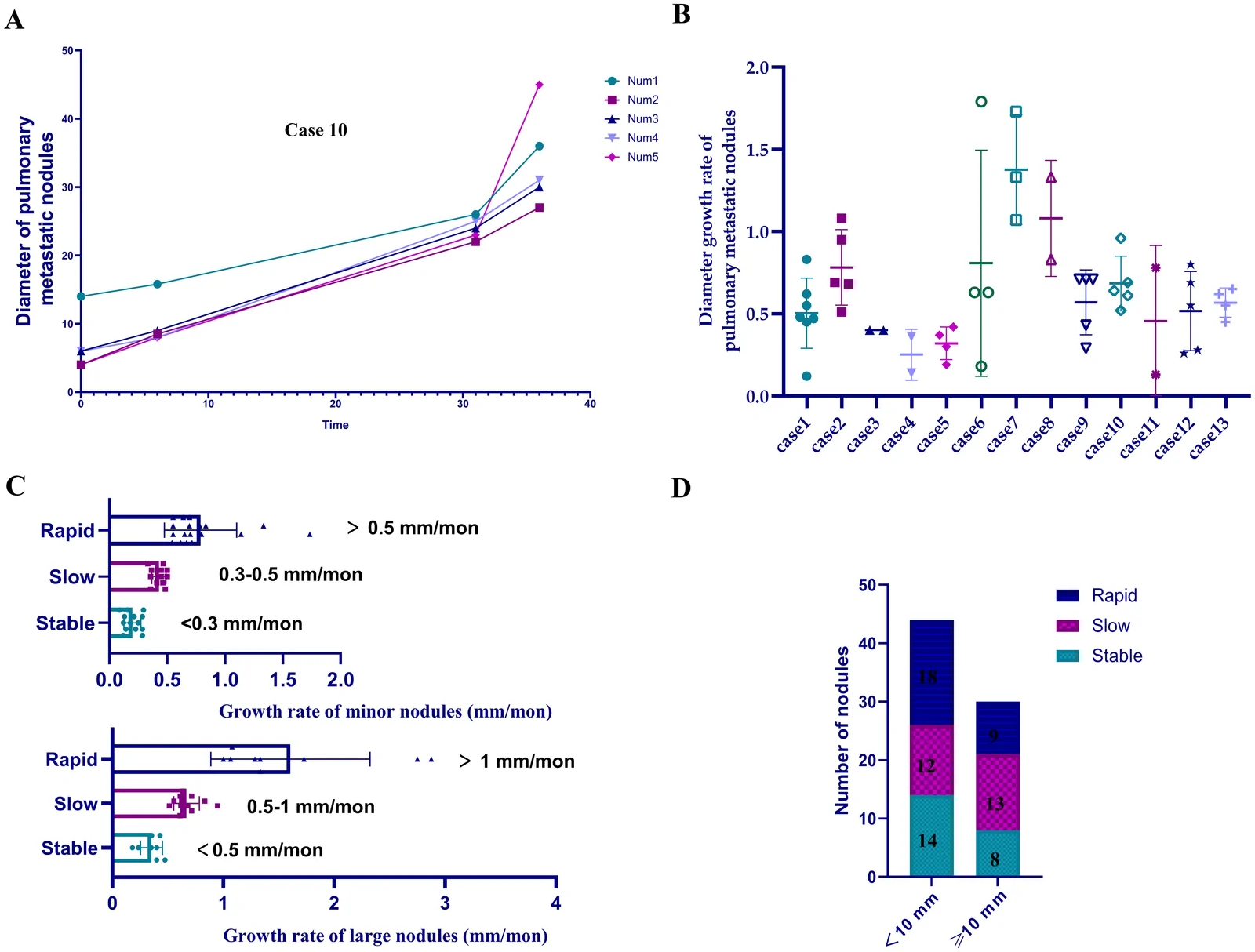
Heterogeneous growth and classification of multiple nodules in lung metastasis. **(A)** demonstrates the diameter growth rate of five lung nodules in Case 10. **(B)** displays the distribution of diameter growth rates in multiple nodules from 13 patients with multiple lung metastases. **(C)** shows the categorization of diameter growth rates in 74 nodules into two groups: the large nodule group and the small nodule group. **(D)** exhibits the distribution of risk subtypes in the large nodule group and small nodule group among the 74 nodules.

After comparing the diameter growth rate of the multiple nodules in the same case, we discovered that only 38.5% (5/13) of patients exhibited consistency in the diameter growth rate of their multiple metastatic lesions. These patients demonstrated minimal numerical fluctuations and a coefficient of variation of less than 30%. In contrast, 61.5% (8/13) of patients showed significant numerical fluctuations, with a coefficient of variation exceeding 30%. Most strikingly, cases 6 and 11 exhibited a coefficient of variation exceeding 80% (**Figure 3B**).

In a subsequent analysis, the 74 nodules in the 37 patients were categorized based on their initial diameter and further stratified by their diameter growth rate. Among the large nodules (≥1mm), 30.0% (9/30) displayed rapid progression with a growth rate beyond 10mm/month, 43.3% (13/30) progressed slowly at a rate of 0.5-1 mm/month, and 26.7% (8/30) remained stable with a rate under 0.5mm/month. Conversely, of the smaller nodules (<1mm), 40.9% (18/44) exhibited rapid progression at a rate exceeding 0.5mm/month, 27.3% (12/44) had a moderate progression rate of 0.3-0.5mm/month, and 31.8% (14/44) were stable, progressing at less than 0.3mm/month, as depicted in **Figures 3C, D**.

### 3.3 Assessment of heterogeneous expression and progressive evolution of metastatic nodules via single-cell sequencing

To elucidate the heterogeneity and progressive evolution of different risk subtypes of lung metastatic nodules, we analyzed the single-cell sequencing data from three multiple nodules of an ACC lung metastasis patient in their natural state (data extracted from a previously published study by our team). The patient’s primary lesion originated from the e epiglottis (T1N0M0) and was treated with surgery and postoperative local radiation. Forty-eight months after the primary surgery, lung nodules were discovered at a local hospital, and at 64 months post-surgery, a right middle and lower lung lobectomy was performed at our institution. From the resected lung lobe specimens, we conducted single-cell sequencing on three lung metastatic nodules: 1) nodule A1 in the right lower lobe (comprising more than 60% solid component); 2) nodule B1 in the right middle lobe (mainly showing tubular pattern); and 3) nodule C1 in the right lower lobe (comprising more than 80% solid component). Subsequently, we collected diameter progression data from lung CT scans in a retrospective manner and determined the diameter progression rates of nodules: A1 at 0.66 mm/month, B1 at 0.13 mm/month, and C1 at 0.65 mm/month. The initial diameters of the three nodules were all smaller than 10 mm, and based on the analysis of diameter progression rates, nodules A1 and C1 were defined as rapid-progressing nodules, while nodule B1 was classified as a stable nodule (**Figures 4A, B**).

**Figure 4:**
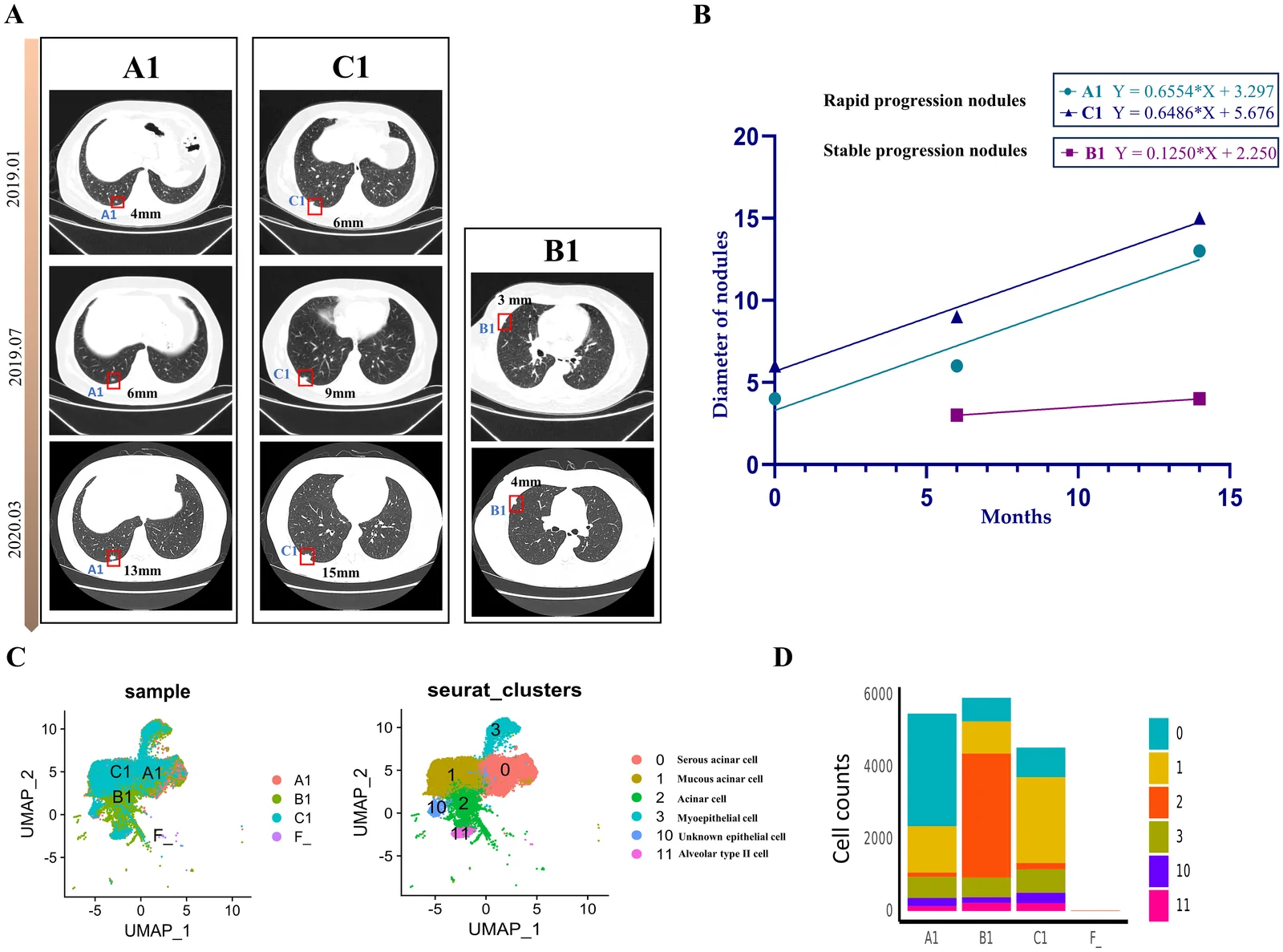
Single-cell sequencing analysis of three lung metastatic nodules. **(A)** Continuous measurements of three lung metastatic nodules' diameters. **(B)** Growth status of the three nodules' diameters. **(C)** UMAP plot showing four lung metastasis samples and six clusters of cancer cells. **(D)** Cell cluster composition plot for the four samples.

Six cancer clusters, defined by previous research, from the three tumor samples were selected for further expression analysis (17). After further selection, the cell counts for each sample were: A1 with 5,476 cells, B1 with 5,915 cells, C1 with 4,533 cells, and F with 16 cells, bringing the total to 15,940 cells (**Figure 4C**). These six cancer cell clusters represented the following cell types: Cluster 0: serous glandular cells; Cluster 1: mucous glandular cells; Cluster 2: Acinar cells; Cluster 3: Myoepithelial cells; Cluster 10:Undetermined epithelial cells; Cluster 11: Type II alveolar epithelial cells; The characteristic genes for each cluster are shown in **Figure 5A**.

**Figure 5:**
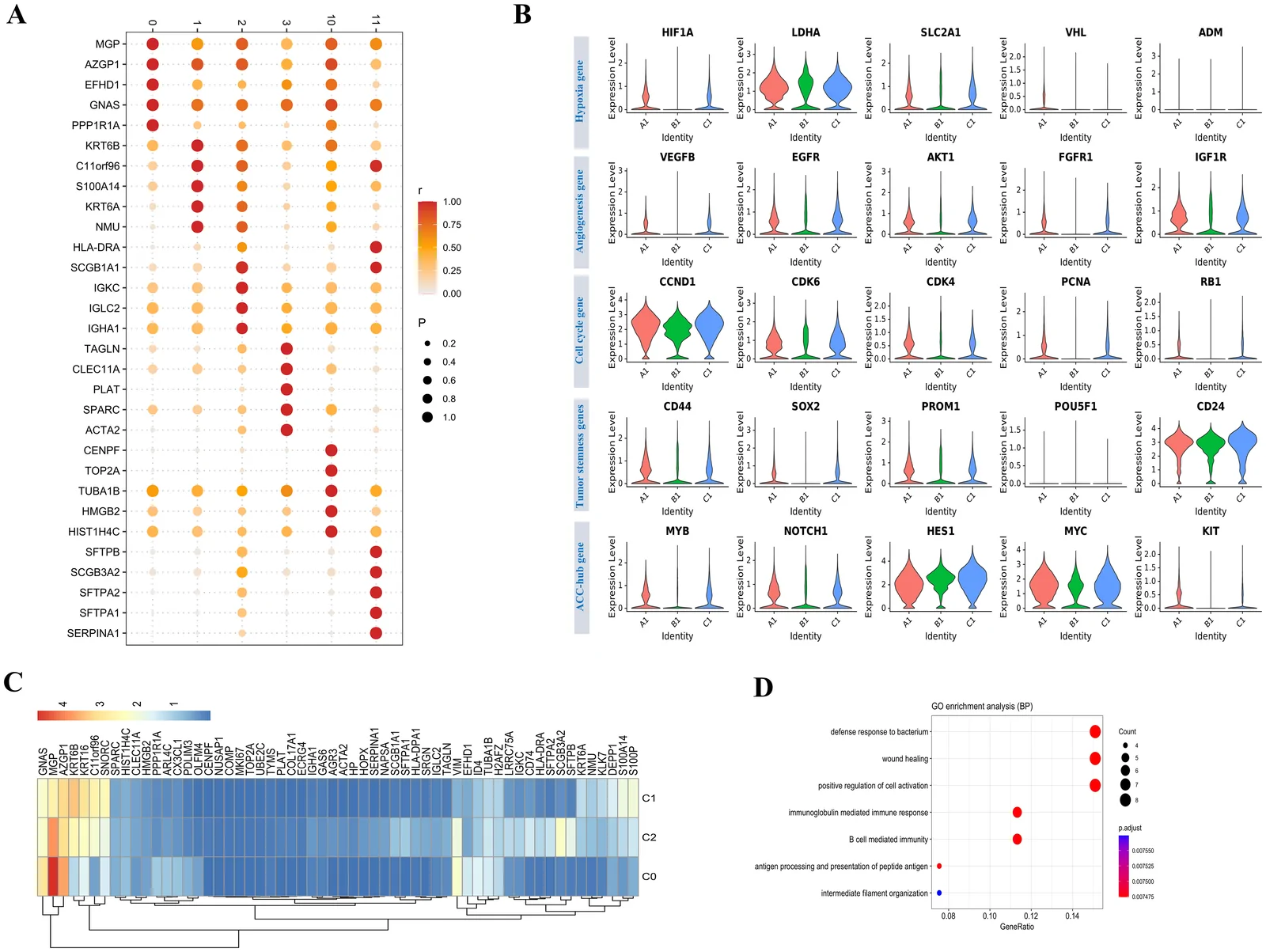
Molecular feature analysis of tumor clusters in lung metastatic nodules. **(A)** Dot plot of marker genes for the six clusters of cancer cells. **(B)** Volcano plot of differentially expressed genes between rapid and stable lung metastasis nodules; **(C)** Differential gene heat map of cluster 2 compared with cluster 0 and Cluster 1; **(D)** Results from GO enrichment analysis for differentially expressed genes in Cluster 2.

In contrast to the stable nodule (B1), the two rapid-progressing nodules (A1 and C1) showed higher expression levels of hypoxia, angiogenesis, cell cycle, and tumor stemness-related factors (**Figure 5B**). Subsequently, we compared the differences in cell composition between different samples and found that in the larger-sized tissues (A1 and C1), Cluster 0 (A1: 0.558, B1: 0.1, C1: 0.174) and Cluster 1 (A1: 0.194, B1: 0.126, C1: 0.462) were more predominant in A1 and C1 samples, while Cluster 2 was significantly higher in B1 sample (A1: 0.021, B1: 0.494, C1: 0.033) (**Figure 4D**). We further compared the differentially expressed genes in Cluster 2 compared to Clusters 0 and 1 and performed the biological process (BP) of Gene Ontology (GO) gene enrichment analysis, which revealed enrichment in immune-related signaling pathways, such as immunoglobulin-mediated immune response and B cell-mediated immunity.

Subsequently, the Monocle tool was used for single-cell trajectory analysis on the three cancer cell clusters 0, 1, and 2, using unsupervised clustering. Highly variable genes were used as sorting genes to order individual cells downstream. Under the default root state value (Null), Cluster 2-dominated state1 was identified as the root state based on their characteristic expression patterns (**Figure 6A**). We screened for differentially expressed genes in the clusters, conducted differential expression analysis of pseudotime, and performed clustering. Down-regulation of factors in immune response-related pathways, such as immunoglobulin-mediated immune response, B cell-mediated immunity, and lymphocyte-mediated immunity, was found (**Figure 6B**). Single-gene pseudotime analysis revealed a gradual increase in Notch1 expression during the progression from Cluster 2 to Clusters 0 and 1. During the evolution from cluster 2 to cluster 0 and cluster 1, the expression of MYB, NFIB, HEY1 and MYC was relatively high in cluster 0 and relatively low in cluster 1, which affected the differentiation of the cluster.

**Figure 6:**
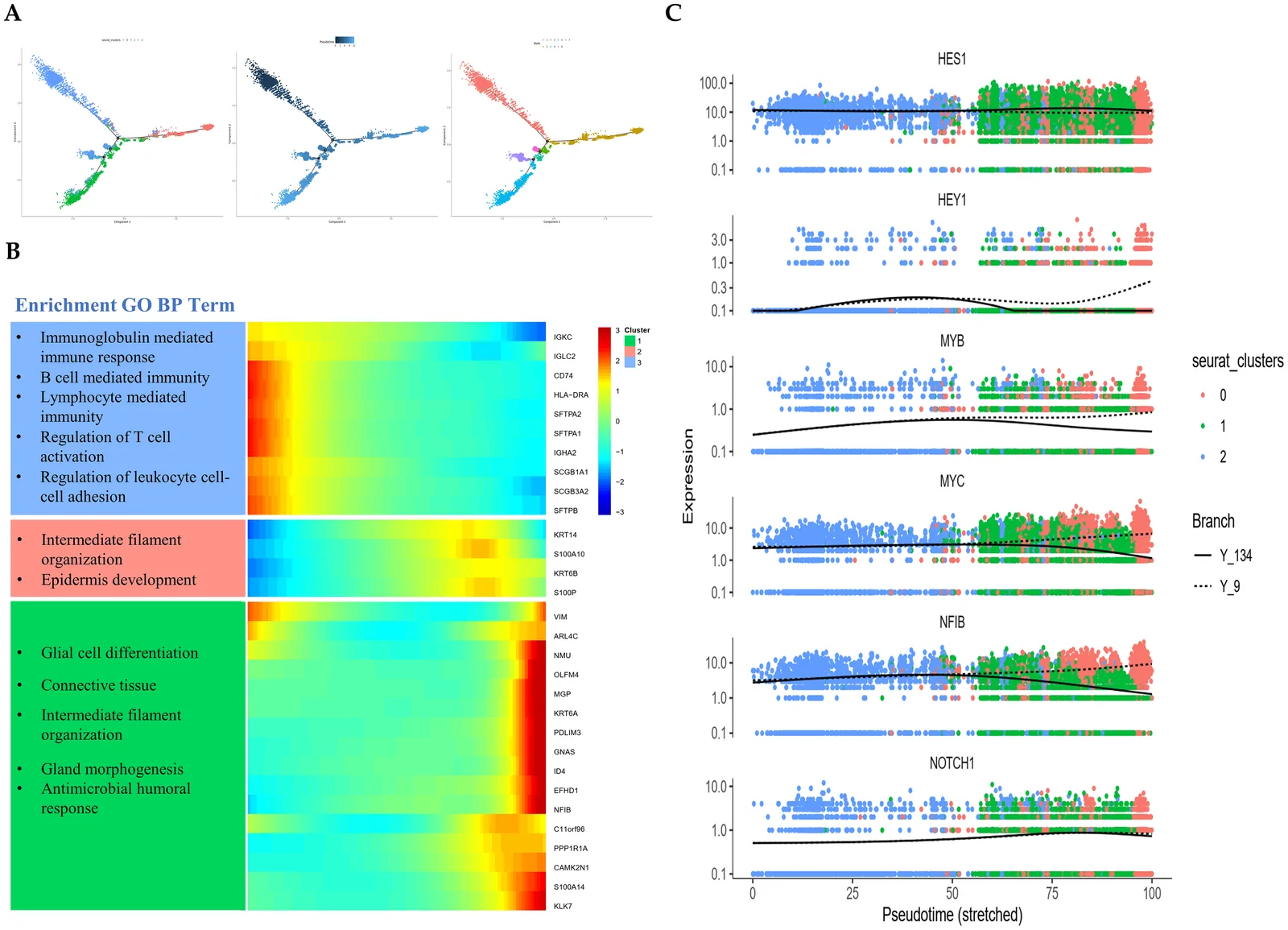
Molecular features of the progressive evolution of lung metastasis identified by the pseudotime analysis. **(A)** The pseudotime analysis of Clusters 0, 1, and 2. The darker color in the top left corner of the intermediate image indicates the starting point of the pseudo-time analysis. With the progression of pseudo-time, seven distinct cell states and three branching points are observed. **(B)** Differentially expressed genes based on pseudo-time values. Genes with the same dynamic trend during pseudo-time are clustered into three groups, and GO (BP) gene enrichment analysis is conducted for each group. Based on the color-coded reference axis, the first group (blue background) demonstrates a gradual decrease in gene expression. **(C)** The pseudo-time analysis of individual genes, depicting the evolution of gene expression during pseudotime and cluster transitions.

## 4 Discussion

This study preliminarily investigated the baseline growth rate characteristics of lung metastatic nodules in patients with head and neck adenoid cystic carcinoma who had not received systemic treatment. By integrating clinical data with single-cell sequencing results, we also preliminarily explored potential factors associated with the progression of metastatic nodules, aiming to provide a reference for the future establishment of clinical stratified management strategies.

In this preliminary study based on a small cohort, our research results suggest that pulmonary metastatic nodules of head and neck ACC may exhibit significantly heterogeneous growth patterns, which can be preliminarily categorized into three types: rapid progression (diameter growth rate > 1 mm/month), slow progression (growth rate between 0.3-1 mm/month), and stable state (growth rate < 0.3 mm/month). Within our study sample, most patients (56.8%) present with slow progression, further suggesting that in the natural disease course, pulmonary metastatic nodules may prosess relatively indolent biological behavior in the natural course of the disease. This finding is consistent with previous reports on the slow-progressing nature of ACC pulmonary metastases, such metastases usually progress slowly or even remain stable for a long time: the doubling time of pulmonary metastatic nodules in adenoid cystic carcinoma ranges from 86 to 1064 days, with an average of 393 days, which is much longer than that of most other malignant tumors (18). This observation may partly explain why some patients can maintain a relatively long treatment-free survival period after being diagnosed with lung metastasis. However, the observed 16.2% of cases with rapid progression suggest the possible existence of a small subset of highly aggressive subpopulations. In these cases, a diameter growth rate exceeding 1 mm per month may be associated with rapid disease deterioration, thereby providing a preliminary quantitative reference for the selection of clinical intervention timing. In conclusion, based on the comparison of diameter growth rates of nodules in their natural state within this limited dataset, we have made an initial attempt to stratify patients based on the progression of lung metastases. We propose that for patients in the early stage of lung metastasis who present with a rapid progression pattern, aggressive therapeutic intervention may need to be considered. Conversely, for patients with a stable pattern, intervention could be deferred, and continued follow-up monitoring is recommended. These findings warrant further validation in larger prospective cohorts.

Moreover, accurately assessing the therapeutic inhibitory effect of drugs on lung metastasis in ACC patients remains a challenge. Clinical trials often rely on traditional indicators such as overall survival(OS) to evaluate drug efficacy. However, some ACC patients may still experience relatively long survival periods even after the occurrence of lung metastasis. For instance, in Ronald H. Spiro’s research, 10% of ACC patients had a survival period longer than 10 years after the occurrence of distant metastasis (8). Therefore, traditional assessment indicators may not sensitively reflect the inhibition of lung metastasis progression by drugs with a short-term follow-up period. Given the limitations of traditional indicators for evaluating the therapeutic effect of drugs on ACC lung metastases, we propose based on the findings of this study, a potential evaluation method. This proposal represents a preliminary hypothesis that requires further verification. Specifically, the inhibitory effect of drugs on lung metastases could potentially be assessed by comparing the diameter growth rate of lung metastatic nodules before and after drug administration, or by referencing the natural progression growth rate of ACC lung metastatic nodules calculated in the present study. These approaches, however, warrant validation in future investigations.

In our exploratory analyses based on a small sample size, further univariate studies suggested that low TP63 expression, high pathological grade (grade III), and initial metastatic nodule diameter > 10 mm may be potential predictors of rapid growth within our cohort, which might provide preliminary guidance for clinical practice. Conversely, these factors were not found to be associated with sex, age, primary tumor site, T stage, N stage, M stage, clinical stage or Ki-67 expression level in our univariate analysis. Based on proteomic analysis, a study by Ferrarotto et al. (19) classified ACC into two subtypes, among which ACC-II shows high TP63 expression and is associated with better patient prognosis, a finding that appears consistent with observations from the present study. The underlying mechanisms by which TP63 downregulation promotes accelerated growth of metastatic lesions remain largely undefined, and warrant further exploration in future studies. Literature review suggests that TP63 plays a pivotal role in ACC development, with its expression levels linked to epithelial differentiation, stemness maintenance, and tumor immune microenvironment modulation.

With regard to epithelial differentiation,ΔNp63α endows tumor cells with migratory potential by inducing a hybrid epithelial-mesenchymal phenotype, which exerts a more potent pro-metastatic effect than complete and sustained epithelial-mesenchymal transition (20). On the other hand, TP63 positively regulates epithelial cell adhesion function. Knockdown of TP63 significantly downregulates adhesion-related genes, resulting in cell detachment from the primary tissue microenvironment and subsequent anoikis. In contrast, overexpression of TAp63γ and ΔNp63α can upregulate adhesion molecules, enhance cellular adhesion capacity and enable tumor cells to resist anoikis (21). In addition, TP63, especially the TAp63 isoform, possesses intrinsic inhibitory effects on tumor cell proliferation. Downregulation of TP63 releases this proliferative suppression, thereby markedly elevating the proliferative activity and accelerating the growth rate of tumor cells within established metastatic lesions. This mechanism is well supported by published evidence. Loss of TP63 facilitates cell cycle progression and accelerates the growth of *in vivo* metastatic tumors via activating MAPK and STAT3 signaling pathways (22).

ΔNp63 is an essential molecule for maintaining the self-renewal capacity of various epithelial tissue stem cells (23). ΔNp63 directly upregulates the expression of the WNT ligand receptor FZD7, thereby activating the WNT signaling pathway, which is critical for epithelial stem cell homeostasis. This indicates that activation of the WNT signaling pathway is a core effector pathway through which ΔNp63 regulates cancer stem cell stemness (24). In addition, the NOTCH signaling pathway exhibits precise functional crosstalk with ΔNp63 to maintain a homeostatic balance between luminal lineage commitment and basal cell fate maintenance. In the human breast cancer MCF7 cell line, overexpression of ΔNp63 significantly enhances cancer stem cell-like phenotypes (25). ΔNp63 also promotes the activation of multiple receptor tyrosine kinases, including the epidermal growth factor receptor (EGFR), whose downstream signaling is essential for cancer stem cell proliferation and survival (26, 27).

TP63 is closely involved in the regulation of the tumor immune microenvironment. Studies have shown that ΔNp63 restricts the viral mimicry response in tumor cells by suppressing the cGAS-STING pathway and type I interferon signaling, thereby repelling immune cell infiltration and remodeling the immune microenvironment in esophageal squamous cell carcinoma (28). Another study reported that Np63 cooperates with the oncogene Ras to upregulate relevant chemokines, recruiting immunosuppressive polymorphonuclear myeloid-derived suppressor cells (PMN-MDSCs) and thereby establishing an immunosuppressive microenvironment (29). Furthermore, in bladder cancer, P63 negatively regulates the expression of interferon-stimulated genes by modulating the long non-coding RNA LEADR, thereby affecting tumor cell sensitivity to immune signals (30).

Most existing literature reports indicate that a higher pathological grade is associated with an increased likelihood of lung metastasis in patients. However, to our knowledge, there is no prior literature reporting the relationship between pathological grade and the growth rate of metastatic lung nodules. In the present study, we observed that pathological grade III was associated with a rapidly progressive type within our cohort. A potential explanation is that high-grade AdCC (grade III) is mainly composed of solid components (accounting for >30%), with cells losing normal differentiation function, exhibiting high malignancy, and showing strong invasiveness. It is possible that the accumulation of malignant mutations at the genetic level of tumor cells (such as MYC amplification) may directly drives the subsequent rapid growth of lung metastatic foci. In addition, the initial nodule diameter showed a positive association with the growth rate (P < 0.05), suggesting that, for lesions with a large initial diameter, the base number of viable tumor cells is generally larger; consequently, even if the proliferation rate of single cells is similar to that of small lesions, the “absolute rate” of overall volume growth (e.g., millimeters increased per month) may still be higher. These lesions may therefore have more active biological characteristics and may require close monitoring or early intervention. These findings are preliminary and require validation in larger, independent cohorts.

Abnormal molecular expression may play a role in determining the difference in lung metastasis progression rates across patients. Current research on molecular aberrations associated with this disease highlights MYB/MYBL1 translocation fusion and Notch1 activating mutations as the most significant oncogenic molecular mechanisms in ACC (31–38). Upregulation of MYC and its target genes, and Notch activating mutations have been defined by Ferrarotto et al. as the unfavorable high-risk subtype (ACC-I), with a median survival period of only 3.44 years (19). High expression of TP63 is considered a favorable molecular characteristic of the low-risk subtype (ACC-II) with a better prognosis (19). In the present study, we also observed that low expression of TP63 at the primary site was associated with an increased growth rate of nodules within our cohort. However, the correlation between the expression of molecules such as MYB and Notch1 and the progression rate of lung metastasis remains to be further explored in future studies. It is regrettable that, to date, current clinical trials exploring targeted therapies, including MYB inhibitors like all-trans retinoic acid, Notch inhibitors like brontictuzumab, multitarget tyrosine kinase inhibitors, and EGFR receptor inhibitors, have not yet shown survival benefits for advanced metastatic ACC patients (39–42). These negative results underscore the need for continued efforts to identify effective therapeutic strategies for this patient population.

Molecular expression heterogeneity may contribute not only to differences in patient-level progression states but also to the divergent progression states of multiple lesions within the same individual. In the present study, based on a limited number of nodules, we compared nodules within the same diameter range and multiple nodules within the same case. These comparisons suggested heterogeneity in the progression of lung metastatic nodules. Some nodules exhibited a phenotype suggestive of high risik with rapid progression, while others displayed a phenotype suggestive of low risk with slow or stable progression. Subsequently, as an exploratory analysis, we analyzed single-cell sequencing data from three lung metastatic nodules with different progression states within a single case to identify potential molecular expression characteristics of nodule subtypes. Our exploratory findings indicated that, within this case, the rapid-progression nodules (A1, C1) showed relatively higher expression of genes associated with hypoxia, angiogenesis, cell cycle, and tumor stemness, as well as overexpression of key ACC driver genes MYB and Notch1, when compared with the stable nodules (B1). Based on these observations, multiple nodules within a single case, each with divergent progression states, may exhibit molecular expression disparity, which could be potentially linked to their initial diameters. It is possible that as diameters expand, molecular traits of the tumor lesions might undergo dynamic reprogramming at assorted stages. Given the exploratory nature of these findings based on a single case, they should be interpreted with caution and require validation in larger cohorts with experimental verification.

Furthermore, to explore preliminarily the molecular characteristics of the progressive evolution of lung metastasis, we analyzed the primary cellular components of lung metastatic nodules in different progression states and performed pseudotime analysis of the corresponding main malignant clusters based on single-cell sequencing data from a single case. In this analysis, Cluster 2 was the main malignant component of the stable nodule B1, while Clusters 0 and 1 were the main malignant clusters of the rapidly progressing nodules (A1, C1). Our exploratory findings suggested that Cluster 2 could represent the starting point of differentiation from which Clusters 0 and 1 may emerge, and that during the putative gradual evolution, there was an observed weakening of immune response-related signals. Single-gene pseudotime analysis indicated a gradual increase in the expression of Notch1. Additionally, the differential gene enrichment analysis between Cluster 2 and Clusters 0 and 1 also highlighted immune response-related signals pathways. These observations may offer a potential explanation for the observed change in the growth rate of nodules in their natural state during follow-up: small nodules with an initial diameter of less than 10 mm displayed a long-term stable state in the early stage; however, after maintaining this state for a certain period, they began to accelerate in growth (as shown in **Figure 2A** for case 3, case 15, etc.). A tentative interpretation is that the early immune microenvironment of stable nodules exhibited strong signals, which may have suppressed tumor growth. Subsequently, as the tumor diameter gradually increased and the immune microenvironment became potentially disturbed and suppressed, the growth rate may have accelerated. Given that these findings are derived from a single case and are exploratory in nature, they should be interpreted with caution and require validation in larger cohorts with experimental verification.

The risk stratification system for adenoid cystic carcinoma (ACC) constructed in this study is mainly intended to break the traditional “one-size-fits-all” clinical management model. It achieves individualized risk stratification of patients through quantitative tumor growth kinetic indicators; for example, it is recommended that high-risk patients shorten the follow-up interval, while intermediate and low-risk patients appropriately extend the follow-up interval. For patients with ACC lung metastases with rapid tumor growth, the oncology radical treatment principle should be prioritized, including extended resection and postoperative adjuvant therapy; in contrast, for patients with lung metastases with slow tumor growth, the function-preserving principle should be given priority to avoid overtreatment and conduct strict observation. In addition, tumor growth kinetic indicators can also serve as an “early warning system” for systemic therapy in ACC patients: high-risk patients should initiate treatment in advance during the rapid proliferation phase to curb metastatic progression and achieve precise treatment. Regarding patient selection for clinical trial enrollment, priority should also be given to patients with faster-growing lung metastases to improve the treatment response rate, reduce invalid enrollment, and lower trial costs.

Regrettably, despite our best efforts to ensure scientific rigor, our study is subject to the following limitations. 1.This was a retrospective analysis, which precluded uniform imaging follow-up intervals for all patients. 2.To maximize the sample size for this rare tumor, we were unable to control for a uniform initial diameter range of pulmonary metastatic nodules. 3.The sample size was small (N = 37), and the study had a single-center, exploratory design, which precluded multivariate analysis and stable effect size estimation. The reported P-values should be interpreted as screening signals for hypothesis generation, and all findings require validation in larger cohorts. 4.Although we included one suitable case for single-cell sequencing, its representativeness is limited, and further validation with an expanded sample size is required. 5.Given the rarity of this disease and our limited sample size, we were unable to establish a precise predictive model for the specific time point of accelerated tumor growth. In future long-term follow-up cohort studies, we plan to collect more dynamic imaging data to further explore reliable predictors for the early identification of tumor growth acceleration. 6.Mixed-effects models or cluster analysis were not used to account for the intrapatient correlation of multiple nodules. Future studies will adopt these methods to improve the scientific rigor and reliability of the findings.

In summary, this retrospective analysis of ACC lung metastasis investigated the diameter growth rate of nodules in their natural state without intervention, compared the progression status across the patient population, and made an initial attempt to achieve risk stratification of lung metastasis based on the observed growth patterns. The present study proposes, as an exploratory concept, a population stratification indicator for assessing the progression rate after the occurrence of lung metastasis. Additionally, through an exploratory integration of single-cell sequencing data from a single case, we observed a weakening of immune response signals during the putative progressive transformation from stable nodules to those with a higher-risk phenotype. These preliminary findings offer theoretical support, albeit limited, for risk stratification and precise intervention in lung metastasis, with the long-term goal of advancing lung metastasis treatment in the future. Based on these observations, we hypothesize that for ACC, immunotherapy might be effective if applied in the early stages of lung metastasis.

## Data Availability

The datasets presented in this study can be found in online repositories. The names of the repository and accession number can be found below:Gene Expression Omnibus (GEO), GSE216852 (https://www.ncbi.nlm.nih.gov/geo/query/acc.cgi?acc=GSE216852).
